# Impact of Xylanase and Glucanase on Oligosaccharide Formation, Carbohydrate Fermentation Patterns, and Nutrient Utilization in the Gastrointestinal Tract of Broilers

**DOI:** 10.3390/ani11051285

**Published:** 2021-04-29

**Authors:** Dimitrios Kouzounis, Jos A. Hageman, Natalia Soares, Joris Michiels, Henk A. Schols

**Affiliations:** 1 Laboratory of Food Chemistry, Wageningen University & Research, Bornse Weilanden 9, 6708 WG Wageningen, The Netherlands; dimitrios.kouzounis@wur.nl; 2 Biometris, Applied Statistics, Wageningen University & Research, Droevendaalsesteeg 1, 6700 AA Wageningen, The Netherlands; jos.hageman@wur.nl; 3 Huvepharma NV, Uitbreidingstraat 80, 2600 Berchem, Belgium; natalia.soares@huvepharma.com; 4 Laboratory for Animal Nutrition and Animal Product Quality (LANUPRO), Department of Animal Sciences and Aquatic Ecology, Ghent University, Coupure Links 653, 9000 Ghent, Belgium; joris.michiels@ugent.be

**Keywords:** feed enzymes, cereal NSP, xylanase, broilers, oligosaccharide MALDI-TOF-MS profile, digestion, prebiotics, arabinoxylan-oligosaccharides

## Abstract

**Simple Summary:**

Fiber-degrading enzymes are commonly used as feed additives in poultry nutrition to offset the anti-nutritive impact of cereal fibers. These enzymes have been associated with improved nutrient digestion and hindgut fermentation of fibers, and ultimately, improved animal growth. Nevertheless, the underlying mechanisms are not fully clear. The aim of this research was to determine the ability of fiber-degrading enzymes to break fibers down to smaller, more easily fermentable structures and evaluate its implications on feed digestion and fermentation in broilers. It was shown that fiber-degrading enzymes released oligosaccharides in the upper gastrointestinal tract in wheat-fed broilers. This coincided with higher short-chain fatty acid production in the ceca and improved nutrient digestion in the small intestine. Such processes were not observed in maize-fed broilers. The higher impact of enzymes in the wheat-based diet is believed to be related to the more complex structure of maize fibers as well as to the higher nutritional value of maize compared to wheat. This study further establishes the beneficial influence of fiber-degrading enzyme supplementation on nutrient and fiber use.

**Abstract:**

This study aimed at determining how the degradation of cereal non-starch polysaccharides (NSP) by dietary enzymes during feed digestion can influence nutrient digestibility and NSP fermentability in broilers. Ninety-six one-day-old male broilers were assigned to 4 different treatments: control and enzyme-supplemented wheat-based (WC, WE) or maize-based (MC, ME) treatments. Enzyme supplementation with endo-xylanase and endo-glucanase occurred from day 20 onwards. On day 28, digesta samples were collected. Nutrient digestibility, NSP recovery, oligosaccharide profile, and short-chain fatty acids (SCFA) content were determined. Enzyme supplementation in WE resulted in a higher starch (3%; *p* = 0.004) and protein (5%; *p* = 0.002) digestion in the ileum compared to WC. Xylanase activity in WE led to in situ formations of arabinoxylan-oligosaccharides consisting of 5 to 26 pentose units in the ileum. This coincided with decreased arabinose (*p* = 0.059) and xylose (*p* = 0.036) amounts in the ceca and higher acetate (*p* = 0.014) and butyrate (*p* = 0.044) formation in WE compared to WC. Conversely, complete total tract recovery of arabinoxylan in MC and ME suggested poor maize NSP fermentability. Overall, enzyme action improved nutrient digestibility and arabinoxylan fermentability in the wheat-based diet. The lower response of the maize-based diet to enzyme treatment may be related to the recalcitrance of maize arabinoxylan as well as to the high nutritive value of maize.

## 1. Introduction

Poultry nutrition is aiming at improving meat production in a cost-effective way while adhering to global strategies, such as animal welfare and reduction in feed antibiotics [1,2]. To that end, the development of appropriate interventions is performed in a collaborative way by producers, industry, and academia. Feed supplementation with enzymes has attracted attention since enzymes active on non-starch polysaccharides (NSP) are claimed to offset the anti-nutritive effect of dietary NSP from cereals and legumes [3,4,5].

NSP are an indispensable part of poultry diets. Once ingested, NSP can be partially soluble or insoluble, depending on their botanical source, chemical structure, chain length, and association degree with the other cell wall components [5,6,7]. Although not digestible by endogenous enzymes, NSP can influence feed use throughout the gastrointestinal tract (GIT) [8,9]. The anti-nutritive effect of soluble NSP (arabinoxylan: AX, β-glucan) has been attributed to their ability to increase digesta viscosity, thereby limiting the diffusion of digestive enzymes and nutrients [10,11,12]. Furthermore, increased digesta viscosity may promote pathogen growth in the GIT [10,13]. In addition, insoluble NSP (AX, cellulose) can limit the accessibility of the host’s enzymes to nutrients enveloped by the cell wall and hinder digestion. NSP can potentially exert prebiotic properties, as they can be fermented by microbiota in the ceca into short-chain fatty acids (SCFA). SCFA can promote gut health and provide additional energy to the host, among others [14,15].

Several animal studies have demonstrated that exogenous NSP-degrading enzymes (NSPases) improved broiler performance [12,16,17,18,19,20,21]. For instance, xylanases (EC 3.2.1.8) are hydrolytic enzymes that split the β-(1→4) bonds between unsubstituted xylosyl residues of the xylan backbone [22]. The enzymatic conversion of AX to oligosaccharides (AXOS) with prebiotic potential finds various applications in the feed and food industry [15,22,23]. Xylanases belonging to the glycosyl hydrolase (GH) families 10 and 11 are widely used to improve animal performance, alongside other NSPases, such as β-glucanases, mannanases, and galactosidases [23,24,25]. Beta-glucanases target β-glucans and cellulose present in cereal, and their application in animal feed historically preceded that of xylanases [4]. The enzymatic depolymerization of soluble AX and β-glucan has been linked with reduced intestinal viscosity and, consequently, improved animal performance [12,26]. Nevertheless, viscosity reduction is not the only mechanism involved.

NSPases have been reported to degrade NSP present within the intact cell wall. Such rupture of the cell wall may improve the digestibility of physically entrapped nutrients [18,27]. Additionally, the solubilization of polymeric AX and the release of arabinoxylan-oligosaccharides (AXOS) by NSPases has been linked with increased SCFA production in the broiler’s ceca and could contribute to the NSP’s prebiotic potential [13,15,28,29]. Yet, direct evidence of the in situ formation of potentially prebiotic AXOS remains elusive. To date, the potential of xylanase and glucanase to release entrapped nutrients and to form prebiotic oligosaccharides is still under investigation [4]. Hence, further research is warranted to understand how the postulated prebiotic formation and encapsulated nutrients release may promote gut health and animal growth.

Therefore, it was hypothesized that dietary supplementation of broilers with carbohydrate-active enzymes would enhance carbohydrate fermentation and nutrient digestion. This research aims at determining the potential of dietary xylanase to form oligosaccharides in the upper gastrointestinal tract of broilers fed wheat- or maize-based finisher diets. We further aim to investigate how the enzymatic degradation of NSP may influence carbohydrate fermentation in the hindgut and nutrient digestion in the small intestine.

## 2. Materials and Methods

### 2.1. Diets

All experimental basal diets were manufactured by Research Diet Services B.V. (Wijk bij Duurstede, The Netherlands), as summarized in Table 1. Acid-insoluble ash (Diamol; Franz Bertram GmbH, Hamburg, Germany) was added as a digestibility marker to the finisher diets.

The finisher diets consisted of two different basal diets (wheat or maize) and were provided in mash form as such (control treatment) or were supplemented with commercially available non-starch polysaccharide-degrading enzymes from *Trichoderma* spp. (Huvepharma NV, Berchem, Belgium) (enzyme treatment). The enzymes present were a GH11 endo-1,4-β-xylanase (EC 3.2.1.8), added at 1500 EPU/kg feed (xylanase activity), and an endo-1,4-β-glucananase, added at 100 CU/kg feed (glucanase activity). EPU is defined as the amount of enzyme, which releases 0.0083 μmol of reducing sugars (xylose equivalent) per minute from oat spelt xylan at pH 4.7 and 50 °C. CU is defined as the amount of enzyme, which releases 0.128 μmol of reducing sugars (glucose equivalents) per minute from barley β-glucan at pH 4.5 and 30 °C. The above combinations resulted in four dietary treatments (DT); wheat control (WC), wheat enzyme (WE), maize control (MC), and maize enzyme (ME). The analyzed xylanase and glucanase activities of the enzyme-containing DT ranged between 1550 and 1740 EPU/kg feed and 190–240 CU/kg feed, respectively. The measured chemical composition of wheat-based and maize-based DT is shown in Table 2.

All reagents used were of analytical grade. The water used throughout laboratory experiments was purified with a Milli-Q Integral 5 (Millipore Corp., Billerica, MN, USA) purification system.

### 2.2. Birds Management and Sample Collection

The study was performed at the facilities of the Laboratory for Animal Nutrition and Animal Product Quality (LANUPRO), Department of Animal Sciences and Aquatic Ecology, Ghent University (Belgium), and was conducted in accordance with the ethical standards and recommendations for accommodation and care of laboratory animals covered by the European Directive 2010/63/EU on the protection of animals used for scientific purposes and the Belgian royal decree KB29.05.13 on the use of animals for experimental studies. Birds were housed in one room throughout the study with 23L:1D and 18L:6D (18L from 4:00 a.m. to 10:00 p.m.) light schedule during day 0–7 and beyond, respectively. Room temperature was 34 °C at setting and linearly decreased to 22 °C by day 28. During the first 5 days, additional infra-red lamp heating (one per pen) was used. Ninety-six (96) one-day-old male broilers (Ross 308) (Vervaeke-Belavi; Tielt, Belgium) were wing-tagged and randomly assigned in two floor pens (48 birds/pen): one receiving wheat-based and one receiving maize-based diets, until day 20 of the experiment. The broilers were vaccinated on the first day of age against Newcastle disease and infectious bronchitis at the hatcheries facilities. At 18 days of age, the vaccination against Newcastle disease was repeated with Nobilis ND Clone 30 by spraying. After arrival, birds were fed the starter diets (day 0–10) and grower diets (day 10–20) ad libitum (Table 1). On day 20, the birds were weighed and allocated according to body weight to pens with a wire floor so that the average body weight of birds in each pen was similar. The treatments (WC, WE, MC, and ME) were assigned to pens following a randomized block design. The blocking factor referred to the spatial organization in the facility. Each treatment consisted of 6 replicate pens, with each pen containing 4 birds. During the adaptation period (day 20–24), the birds received the finisher diets ad libitum. The birds were weighed in the morning of day 24 and then continued to be fed finisher diets until day 28. Feed intake was measured per pen and daily (morning of day 25, 26, 27, and 28). During this period, excreta were collected twice daily, homogenized, and an aliquot of a minimum of 250 g fresh material per pen was immediately stored at −20 °C. On day 28, all birds were weighed and euthanized by cervical dislocation followed by bleeding. The gizzard, ileum, and ceca contents were collected, pooled per pen, and frozen at −20 °C. Thawed aliquots were used for the determination of dry matter, ash, and acid-insoluble ash content. Frozen digesta were dried by lyophilization and homogenized with a MM 400 Mixer Mill (Retsch GmbH, Haan, Germany) prior to other chemical analyses. Feed samples were ground to pass a 0.7 mm sieve using a ZM200 mill (Retsch GmbH) prior to analysis.

### 2.3. Proximate Composition Analysis

#### 2.3.1. Dry Matter, Ash, and Acid-Insoluble Ash Content

Feed samples and thawed aliquots from the gizzard, ileum, and excreta were dried in an air oven at 80 °C, overnight. Subsequently, the dry matter content was determined by drying at 103 °C, according to the AOAC 935.29 method [30]. For that purpose, approximately 5 g feed samples and 1–2 g digesta were weighed in ceramic crucibles. Next, ash and acid-insoluble ash (AIA) contents were determined sequentially, according to the method described by Van Keulen and Young (1977) [31] with certain modifications introduced by Montaño-Vargas et al. (2002) [32], allowing the reduction in sample size. In brief, dried samples were incinerated at 575 °C, and the resulting ash was weighed and boiled with 10 mL 4 N HCl and filtered through ashless filter paper. The retentate was incinerated again at 575 °C, and the remaining AIA was weighed. The organic matter (OM) was calculated by subtracting ash from dry matter.

#### 2.3.2. Cecal Dry Matter

Due to sample limitations, the dry matter and ash content of cecal digesta were determined gravimetrically using an XP6 Excellence Plus Micro Balance (5 decimals) Mettler-Toledo International Inc., Columbus, OH, USA). Approximately 2 mg of fresh cecal matter was weighed in Eco-Cup LF pyrolysis cups (Frontier Laboratories Ltd., Fukushima, Japan) and were incubated at 80 °C, overnight. Next, the samples were incubated at 103 °C for 4 h and weighed. The ash content was determined by incinerating the dried samples at 575 °C and weighing the remaining material.

#### 2.3.3. Crude Protein Content

The nitrogen content of feed samples and digesta was determined according to the AOAC 990.03 method [30] using a FlashEA^®^ 1112 NC Analyzer (Thermo Fisher Scientific Inc., Waltham, MA, USA). The nitrogen conversion factor used to estimate the crude protein was 6.25.

### 2.4. Carbohydrate Analysis

#### 2.4.1. Sugar Composition

The total sugar composition of feed and digesta samples was determined according to Englyst and Cummings (1984) [33]. Samples were pre-hydrolyzed in 72% (*w*/*w*) H_2_SO_4_ (30 °C, 1 h) and subsequently hydrolyzed in 1 M H_2_SO_4_ (100 °C, 3 h). Neutral monosaccharides released were derivatized to alditol acetates and analyzed by gas chromatography on a Trace 1300 GC system (Thermo Fisher Scientific Inc.) equipped with a DB-225 column (Agilent Technologies Inc., Santa Clara, CA, USA) and a flame/ionization detector (FID), using inositol as internal standard. Uronic acid content was determined by the colorimetric *m*-hydroxyphenyl assay with an automated analyzer (Skalar Analytical B.V., Breda, The Netherlands), according to Blumenkrantz and Asboe-Hansen (1973) and Thibault and Robin (1975) [34,35].

#### 2.4.2. Total Starch Content

The total starch content of feed and digesta samples was determined according to the AOAC Method 996.11 (KOH format) [36] using the Total Starch Assay Kit (Megazyme, Bray, Ireland) as modified by Martens et al. (2018) [37]. In brief, 25 μL supernatant of enzymatically treated samples was transferred in the wells of a 96 well plate followed by the addition of 225 μL glucose oxidase peroxidase (GOPOD) reagent (Megazyme). The reaction was performed in a shaking incubator at 50 °C for 20 min, and the absorbance at 520 nm was read against reagent blank using a Tecan Infinite^®^ F500 (Tecan Group Ltd., Männedorf, Switzerland) spectrophotometer. The glucose (Glc) content was determined using a Glc calibration curve (0.1–0.6 mg/mL).

#### 2.4.3. Oligosaccharide Characterization by Matrix-Assisted Laser Desorption/Ionization Time-of-Flight Mass Spectrometry (MALDI-TOF-MS)

The structural characterization of oligosaccharides present in the ileum was performed according to Broxterman et al. (2017) [38] on an ultrafleXtreme^TM^ MALDI-TOF/TOF mass spectrometer (Bruker Daltonics Inc., Billerica, MA, USA). The equipment was controlled with FlexControl 3.3 software and operated in positive mode. The mass spectrometer was calibrated with maltodextrins (Avebe, Veendam, The Netherlands) in a mass range of 500–3000 (*m*/*z*). Approximately 100 mg of dried ileal digesta was suspended in 1 mL water and incubated at 99 °C for 30 min. The supernatant was then separated by centrifugation at 20,000× *g* for 10 min, diluted ten times with water, and desalted with Dowex 50W-X8 resin (Sigma-Aldrich, St. Louis, MO, USA). Next, an aliquot (100 μL) was removed, and NaCl was added at 1 μM to allow the formation of sodium adducts during ionization. Afterward, sample (1 μL) was co-crystallized with matrix solution (1 μL); 25 mg/mL dihydroxy-benzoic acid (Sigma-Aldrich) in 50% (*v*/*v*) acetonitrile (VWR International B.V., Amsterdam, The Netherlands) on a target plate under a stream of dry air.

### 2.5. Microbial Metabolites Analysis

#### 2.5.1. Short-Chain Fatty Acids (SCFA)

The SCFA content of ileal and cecal digesta was determined by gas chromatography (GC-FID), as described by Logtenberg et al. (2020) [39]. An aqueous solution of acetic, butyric, propionic, isobutyric, and isovaleric acids (Sigma-Aldrich) was prepared for quantification. The standard solution was diluted to obtain final concentrations in the range of 0.01–1.0 mg/mL and was treated similarly to the samples.

#### 2.5.2. Lactic and Succinic Acids

The concentrations of lactic and succinic acids in ileal and cecal samples were determined by high-performance liquid chromatography (HPLC), according to Jonathan et al. (2012) [40]. The samples were analyzed with an Ultimate 3000 HPLC System (Dionex Corp., Sunnyvale, CA, USA) equipped with an Aminex HPX-87 H column (Bio-Rad, Richmond, VA, USA) and a guard column. The HPLC system was coupled to a Shodex RI-101 refractive index detector (Showa Denko KK, Kawasaki, Japan). The samples (injection volume 10 μL) were run isocratically using 5 mM H_2_SO_4_ as eluent at 0.6 mL/min flow rate, with column temperature at 40 °C. A standard solution containing lactic and succinic acid (Sigma-Aldrich) was prepared for quantification and was diluted to obtain final concentrations in the range of 0.1–10.0 mg/mL.

### 2.6. Calculations

The apparent ileal digestibility (AID) and apparent total tract digestibility (ATTD) of organic matter, starch, and protein were estimated with Equation (1), using AIA as an indigestible marker [20]:

AID or ATTD (%) = ((NT_d_/AIA_d_) − (NT_i,e_/AIA_i,e_))/(NT_d_/AIA_d_) ∗ 100 (1)

where NT_d_, NTi, NT_e_ is the measured nutrient content (% DM) in the diet, ileum, and excreta, respectively, and AIA_d_, AIA_i_, AIA_e_ is the measured marker content (% DM) in the diet, ileum, and excreta. NT_i_ and AIA_i_, and NT_e_ and AIA_e_ were used to determine AID and ATTD, respectively.

The recovery of NSP in the ileum and excreta was determined through the constituting monosaccharides. For that reason, the recovery of xylose, arabinose, galactose, uronic acid, and non-glucosyl NSP (NGP) was estimated using Equation (2):

Recovery (%) = 100 − ((M_d_/AIA_d_) − (M_i,e_/AIA_i,e_))/(M_d_/AIA_d_) ∗ 100 (2)

where M_d_, Mi, M_e_ is the measured monosaccharide content (% DM) in the diet, ileum, and excreta, respectively, and AIA_d_, AIA_i_, AIA_e_ is the measured marker content (% DM) in the diet, ileum, and excreta. M_i_ and AIA_i_, and M_e_ and AIA_e_ were used to determine recovery in the ileum and excreta, respectively.

### 2.7. Statistical Analysis

The obtained data were subjected to analysis of variance (ANOVA) using the R statistical software (R Core Team), with the pen being the experimental unit. The observations from wheat-based and maize-based DT were modeled separately. The effect of enzyme treatment (E; control vs. enzyme) on carbohydrate content and microbial metabolites was determined. Nutrient digestibility and NSP recovery were modeled using E and Sampling Site (S: ileum for AID and excreta for ATTD) as main effects, including their two-way interaction term. The blocking factor was considered as the main effect in the model. To test the significance of the differences between different treatments, Tukey’s post-hoc test was performed, with a significance threshold set at *p* < 0.05.

The data obtained for NSP content and recovery along the GIT, SCFA content in the ceca, nutrient digestibility, and animal growth were subjected to principal component analysis (PCA) using R. Next, the Pearson correlation coefficients of the aforementioned variables were calculated, and the corresponding correlation matrix was constructed to visualize the relations. Correlations with *p* < 0.05 were considered significant.

## 3. Results

### 3.1. Growth Parameters

The growth of broilers was recorded during the finisher period (day 24–28) in order to evaluate the possible effect of enzyme addition on the broiler’s nutritional responses (Table A1). It should be noted that the first aim of this experiment was not to evaluate the effect of the enzyme on animal performance. Therefore, the measured growth parameters are approached with caution and only considered in the context of this study. The obtained values for body weight (BW), average daily gain (ADG), and average daily feed intake (ADFI) were lower, and the feed conversion ratio (FCR) was higher than breed performance objectives for 28-day-old male Ross 308 broilers [41], mainly because broilers were fed mash diets. Overall, WE presented increased BW (6% higher; *p* = 0.021), ADG (14% higher; *p* = 0.059) and ADFI (6% higher; *p* = 0.281) values compared to WC, while FCR decreased by 7% (*p* = 0.018). ME presented numerically positive responses compared to MC, but not to the extent observed in the wheat-based DT. For example, ME presented increased BW (3% higher; *p* = 0.136), ADG (6% higher; *p* = 0.320) and ADFI (4% higher; *p* = 0.285) values compared to MC, while FCR was decreased by 2% (*p* = 0.498).

### 3.2. Oligosaccharide Profiles in Ileal Digesta

**Figure 1:**
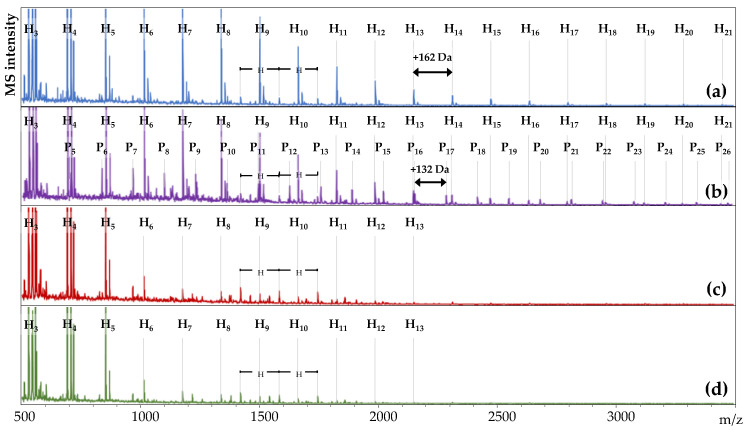
MALDI-TOF-mass spectra of supernatants of ileal digesta from broilers fed wheat control (**a**), wheat enzyme (**b**), maize control (**c**), and maize enzyme (**d**) dietary treatments (DT). The number of hexose (H_n_) or pentose (P_n_) monomers constituting each oligomer is presented in the mass spectra.

**Figure A1:**
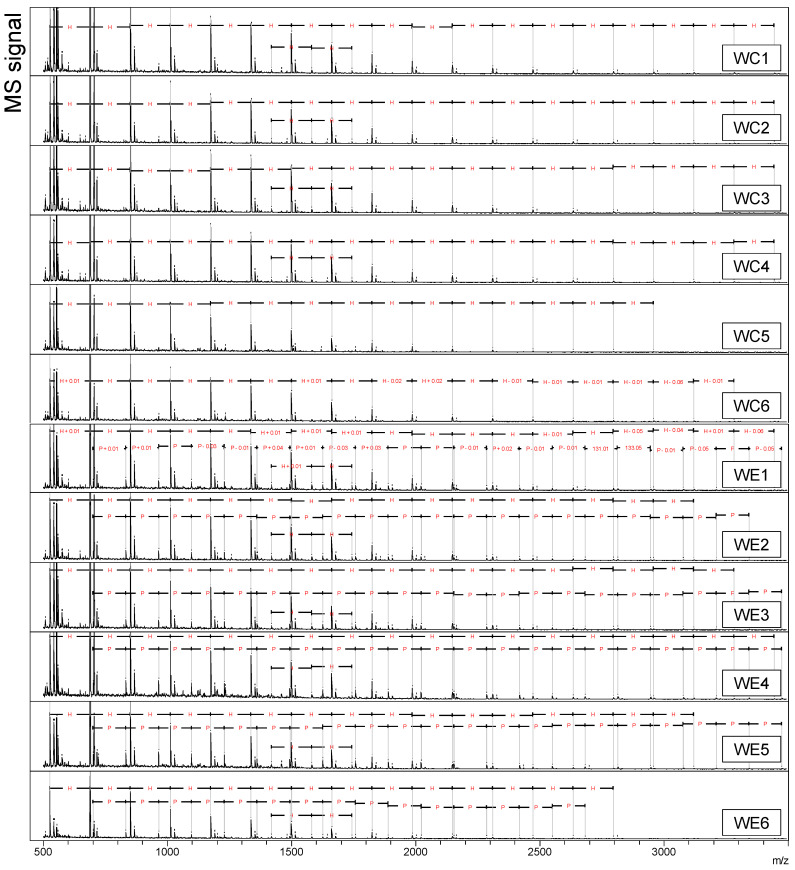
MALDI-TOF-mass spectra of the individual replicates (1–6) of ileal digesta from broilers fed the wheat control (WC) and wheat enzyme (WE) DT. Oligosaccharides consisting of hexose (H; *m*/*z* 162) or pentose (P; *m*/*z* 132) units are shown.

**Figure A2:**
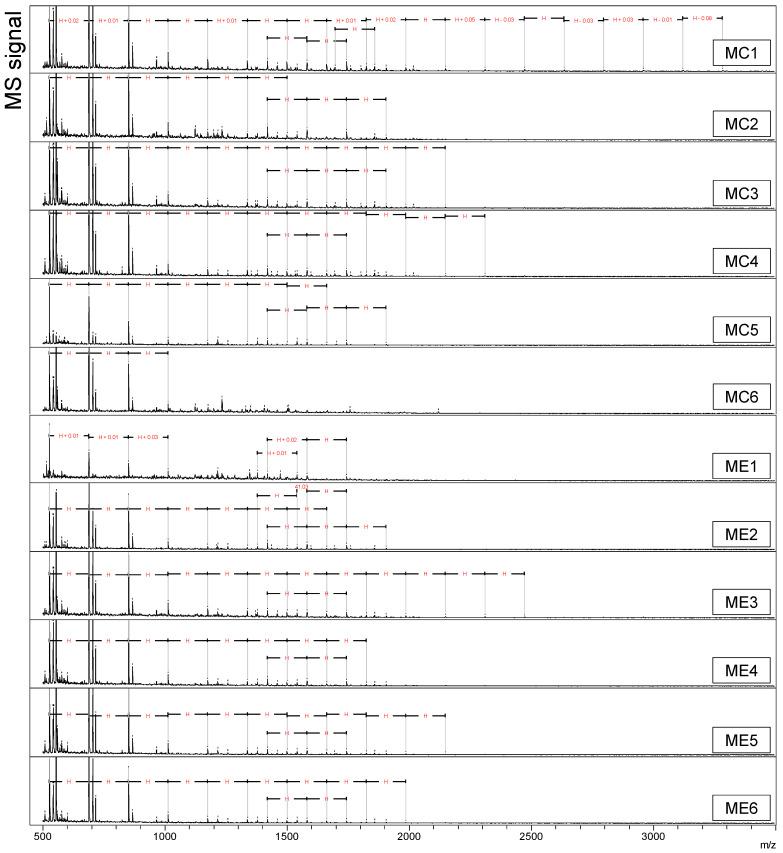
MALDI-TOF-mass spectra of the individual replicates (1–6) of ileal digesta from broilers fed the maize control (MC) and maize enzyme (ME) DT. Oligosaccharides consisting of hexose (H; *m*/*z* 162) units are shown.

The addition of dietary xylanase is hypothesized to degrade polymeric arabinoxylan (AX) to oligosaccharides (AXOS) during feed digestion, and these products are expected to be released in solution. The ability of dietary xylanase to form oligosaccharides was determined by MALDI-TOF-MS analysis of the water-soluble fraction of ileal digesta from the four DT (Figure 1, Figure A1 and Figure A2).

At first glance, *m*/*z* values corresponding to homologous series of hexose oligomers were abundantly detected in all samples (sequential increments of *m*/*z* 162). The hexose oligomers in both wheat-based DT had a polymerization degree (DP) of 3 to 21 (Figure 1a,b). Hexose oligomers of DP 3 to 10 were detected in both maize-based DT (Figure 1c,d). Another series of three oligomers with two consecutive *m*/*z* 162 increments (*m*/*z* 1419, 1581, and 1743) was present in all four DT. Alongside these compounds, a homologous series with increments of *m*/*z* 132 was detected in WE, representing pentose oligomers (Figure 1b). The pentose oligomers were detected in all six replicate pens (Figure A1) between *m*/*z* 701 and *m*/*z* 3444 and presented DP 5–26. The pentose oligomers were unique for the WE treatment and were absent in WC, MC, and ME.

### 3.3. Monosaccharide Contents in Digesta

In order to evaluate the effect of enzyme addition on the carbohydrate content present in digesta, the monosaccharide content after acid hydrolysis of all carbohydrates present in the finisher diets, gizzard, ileum, ceca, and excreta was determined (Table 2, Table 3 and Table A2). Glucose (Glc) was the most abundant monosaccharide in all diets, followed by xylose (Xyl), arabinose (Ara), galactose (Gal), and uronic acids (UA) (Table 2). Mannose (Man), rhamnose (Rha) and fucose (Fuc) were present in the diets at values lower than 0.6%, 0.1% and 0.2% (*w*/*w*), respectively (data not shown). Man, Rha and Fuc were taken into account when estimating the total carbohydrate contents but will not be further discussed due to their low amounts.

Gizzard: Glc was the main carbohydrate present in the gizzard and ranged between 33.7% (*w*/*w*) and 38.5% (*w*/*w*) (Table 3). In wheat-based DT, Xyl was the second most abundant carbohydrate (7.8–8.9% (*w*/*w*)), followed by Ara, Gal and UA. WC presented significantly higher Glc content than WE (*p* = 0.014). At the same time, WC presented lower Ara (*p* = 0.043), Xyl (*p* = 0.051), Gal (*p* = 0.001), UA (*p* = 0.010) and non-glucosyl NSP (NGP) (*p* = 0.013) contents than WE. No differences in total carbohydrates (*p* = 0.203) and A/X ratio (*p* = 0.230) were observed between WC and WE. MC and ME presented similar monosaccharide contents in the gizzard (*p* > 0.05).

Ceca: The Xyl content in the ceca significantly decreased upon enzyme addition (*p* = 0.036), from 0.6% (*w*/*w*) in WC to 0.2% (*w*/*w*) in WE. The Ara content showed a trend to decrease upon enzyme addition (*p* = 0.059) from 0.3% (*w*/*w*) in WC to 0.2% (*w*/*w*) in WE. The decrease in Ara and Xyl contents coincided with a significantly higher A/X ratio in WE (1.11) compared to WC (0.64) (*p* = 0.005). The Xyl content in the ceca for the maize-based DT was found to be lower than 0.1% (*w*/*w*), while higher A/X values (MC: 2.41, ME: 2.52) than in the wheat-based DT were obtained. ME presented significantly lower NGP content than MC (*p* = 0.036). It should be mentioned that the cecal samples contained 1.1–1.3% (*w*/*w*) rhamnose (data not shown). Since this monosaccharide was only present in trace amounts in the diets, it is suspected to originate from the bacterial cell wall.

Ileum and excreta: Carbohydrates accounted for approximately 44.9–48.8% (*w*/*w*) of the solids present in the ileum (Table A2). Glc was the most abundant carbohydrate, followed by Xyl, Gal, Ara, and UA. The carbohydrate content in the excreta was somewhat lower than in the ileum (35.0–37.1% (*w*/*w*)) (Table A2). Glc was the most abundant carbohydrate, followed by Xyl, Ara, Gal, and UA. To further investigate the transit and fermentability of NSP and individual polymers in the GIT, the recovery values of individual carbohydrates in the ileum and excreta (Equation (2)) were determined and are shown next.

### 3.4. Recovery of NSP in the Ileum and the Total Tract

The transit and fermentability of NSP and individual polymers in the GIT were studied by estimating the recovery values of Ara, Xyl Gal, UA, and NGP in the ileum and excreta (total tract) (Table 4). The absence of significant interactions between enzyme (E) and sampling site (S: ileum, total tract) suggested that the effect of the enzyme was independent of the sampling site for both wheat-based and maize-based DT.

In the wheat-based DT, the sampling site significantly influenced the recovery values of all measured monosaccharides (*p* < 0.05). Significantly lower values were obtained in the total tract compared to the ileum in all cases (*p* < 0.05). Enzyme addition significantly affected the recovery of Ara (*p* = 0.022) and Xyl (*p* = 0.025). Ara recoveries in the ileum were close to 100% for both WC and WE. Furthermore, 94.8% and 88.5% of the Xyl present in the diet was recovered in the ileum for WC and WE, respectively. The differences observed between WC and WE regarding the Ara and Xyl recovery in the ileum were not significant (Ara: *p* = 0.130, Xyl: *p* = 0.214). Nevertheless, the Ara and Xyl values in WE tended to be lower than in WC by 6.3% and 6.7%, respectively. Similarly, the total tract recoveries of Ara and Xyl obtained in WE tended to be lower than the ones obtained in WC (4.3% and 5.9% lower, respectively) but not significantly different (*p* = 0.459 and *p* = 0.628, respectively).

In maize-based DT, the sampling site significantly influenced the Gal (*p* < 0.001) and NGP (*p* = 0.011) recovery values, with lower values being obtained in the total tract compared to the ileum. On the contrary, there was no significant effect of sampling site on Ara (*p* = 0.743), Xyl (*p* = 0.111) and UA (*p* = 0.912) recovery. The ileal and total tract recoveries of Xyl, Ara, and UA were similar, fluctuating around 100% of the constituent monosaccharides present in the diet. No significant effect of enzyme addition was observed in all cases (*p* > 0.05).

### 3.5. Lactate, Succinate, and Short-Chain Fatty Acids (SCFA) Contents in the Ileum and the Ceca

The formation of lactate, succinate, and SCFA in the broiler’s ileum and ceca was determined to monitor the effect of enzyme supplementation on the fermentation processes along the GIT (Table 5). Lactate was the most abundant metabolite in the ileum (129.4–250.2 μmol/g dry matter basis), while acetate and succinate contents ranged between 2.5 and 9.9 μmol/g. Acetate (172.7–354.5 μmol/g) and butyrate (53.1–78.5 μmol/g) were the two most abundant SCFA in the ceca, followed by propionate (11.0–31.4 μmol/g). Isobutyrate and isovalerate were detected in the ceca in considerably lower amounts (1.3–4.3 μmol/g) for all DT.

In the wheat-based DT, enzyme addition significantly increased acetate (*p* = 0.004) and succinate (*p* = 0.013) contents in the ileum. However, it did not significantly affect lactate contents (*p* = 0.163), even though the value obtained in WE was 1.9 times higher than in WC. The reason behind the lack of significance could be the high variation in individual values. Furthermore, enzyme addition significantly increased the contents of acetate (*p* = 0.014), butyrate (*p* = 0.044) and total SCFA (*p* = 0.019) in the ceca. No significant influence of enzyme addition was observed in the contents of propionate (*p* = 0.906), isobutyrate (*p* = 0.728) and isovalerate (*p* = 0.881).

In the maize-based DT, enzyme addition showed a trend to decrease lactate formation in the ileum (*p* = 0.057), but did not impact acetate (*p* = 0.195) and succinate (*p* = 0.425) contents. In the ceca, enzyme addition was found to significantly decrease the contents of acetate (*p* = 0.037), butyrate (*p* = 0.010), propionate (*p* = 0.039) and total SCFA (*p* = 0.021), while it did not impact the contents of isobutyrate (*p* = 0.185) and isovalerate (*p* = 0.333).

Overall, enzyme addition was found to impact the bacterial metabolite formation differently in wheat-based and maize-based DT, highlighting the importance of the cereal type present for hindgut fermentation.

### 3.6. Nutrient Digestibility

The impact of enzyme action on nutrient (organic matter: OM, starch, and crude protein: CP) digestion in the small intestine and OM and starch fermentation in the hindgut is presented in Table 6. The apparent ileal digestibility (AID) values obtained were between 72.2 and 75.3% for OM, 94.8–97.5% for starch, and 77.0–81.2% for CP. The apparent total tract digestibility (ATTD) values obtained ranged between 73.3 and 75.4% for OM and 96.0–98.2% for starch.

A significant enzyme (E) and sampling site (S) interaction (*p* = 0.036) was observed only for OM in the wheat-based DT. Firstly, the pair-wise comparison between the OM-AID and ATTD values of WC and WE revealed that WC-ATTD was significantly higher than WC-AID (*p* = 0.014). However, similar values between WE-AID and WE-ATTD were obtained (*p* = 0.995). Secondly, WE-AID was significantly higher than WC-AID (*p* < 0.001). Lastly, WE-ATTD showed a trend to be higher than WC-ATTD (*p* = 0.074).

Enzyme (E) significantly impacted starch digestibility (*p* = 0.001). In particular, starch WE-AID was significantly higher than WC-AID (*p* = 0.004). No significant differences were found between WE-AID and WE-ATTD (*p* = 0.998). Similarly, no significant differences were found between WC-AID and WC-ATTD (*p* = 0.255). Lastly, WC-ATTD was similar to WE-AID (*p* = 0.185) and WE-ATTD (*p* = 0.248) as well. The similarity of WE-AID and WE-ATTD with WC-ATTD, but not with WC-AID, suggests that the non-significant increase of 1.3% as observed in starch digestibility for WC between the ileum and the total tract could have biological relevance. Finally, enzyme addition significantly increased CP-AID (*p* = 0.002). The nitrogen content in excreta was not corrected for endogenous secretions, and the CP ATTD values were not estimated.

No significant E × S interactions were observed in the maize-based DT (*p* > 0.05). Moreover, enzyme addition did not affect significantly OM (*p* = 0.757), starch (*p* = 0.384) or CP (*p* = 0.102) digestibility. Although the sampling site had a significant effect on OM (*p* = 0.033) digestibility, the individual AID and ATTD values of MC and ME were similar (*p* > 0.05). Next, starch digestibility was significantly affected by the sampling site (*p* = 0.001), with higher values being obtained in the total tract compared to the ileum. In particular, MC-ATTD was significantly higher than MC-AID (*p* = 0.032). ME-AID was not significantly different from MC-AID (*p* = 0.766), but at the same time was similar to both MC-ATTD (*p* = 0.242) and ME-ATTD (*p* = 0.148) values. Hence, a subtle improvement in starch AID due to enzyme addition in maize-based DT could still be of biological importance.

### 3.7. Interrelationships between Nutrient Digestibility, NSP Fermentation in the Hindgut and Growth Parameters

**Figure 2:**
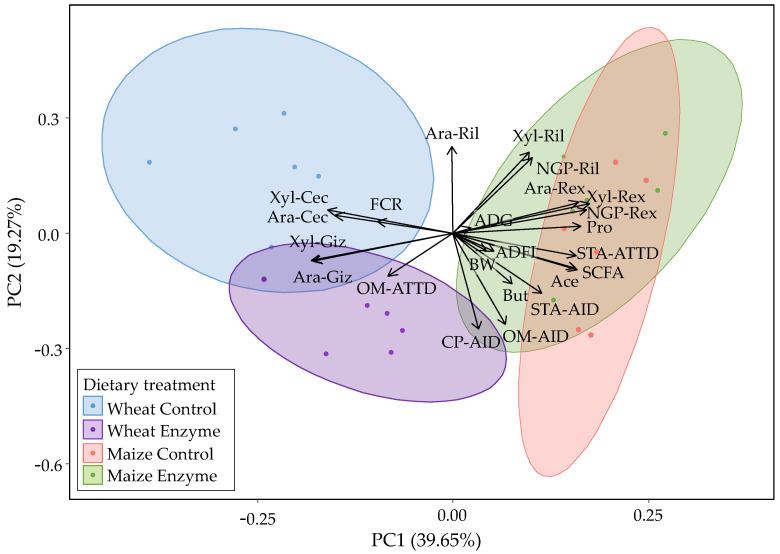
Principal component analysis (PCA) biplot of wheat control (blue), wheat enzyme (purple), maize control (red), and maize enzyme (green) dietary treatments (DT). The scores were plotted for PC1 and PC2. The amount of variance explained by each PC is shown in parentheses. The variables used were: (i) NSP-related parameters; ileal (Ril) and total tract (Rex) recovery of Ara, Xyl and non-glucosyl NSP (NGP), and Ara and Xyl contents (% *w*/*w*) in the gizzard (Giz) and the ceca (Cec), (ii) acetate (Ace), butyrate (But), propionate (Pro) and total short-chain fatty acids (SCFA) contents in the ceca, (iii) nutrient digestibility parameters: apparent ileal digestibility (AID) and apparent total tract digestibility (ATTD) of organic matter (OM) starch (STA), and crude protein (CP), and (iv) animal growth parameters: body weight (BW), feed conversion ratio (FCR), average daily feed intake (ADFI), and average daily gain (ADG).

Principal component analysis was performed to obtain an overview of the response of the different dietary treatments to the investigated parameters (Figure 2).

The first principal component (PC1) explained 39.65%, and the second principal component (PC2) explained 19.27% of the total variance. Overall, PC1 appeared to separate the wheat-based from the maize-based DT while WC and WE were further separated by PC2. WC presented high Ara and Xyl contents in the cecum (Ara-Cec, Xyl-Cec) and high FCR values. WE formed a separate cluster from WC mainly due to the higher Xyl-Giz, Ara-Giz, OM-AID, OM-ATTD, and CP-AID loadings. The maize-based DT were clustered together and presented differences compared to both WC and WE. Both MC and ME were characterized by high Ara, Xyl, and NGP recovery in the ileum (Ril) and excreta (Rex).

**Figure A3:**
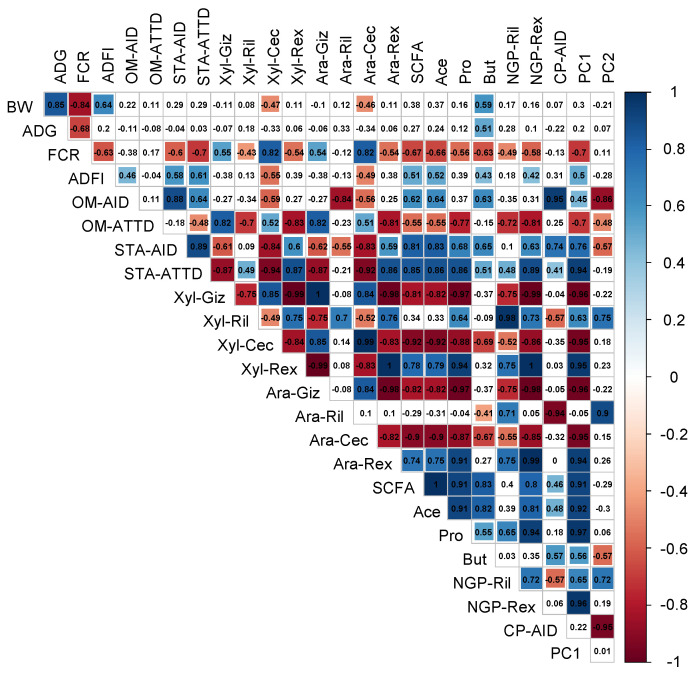
Pearson coefficients between: (i) NSP-related parameters; ileal (Ril) and total tract (Rex) recovery of Ara, Xyl, and non-glucosyl NSP (NGP), and Ara and Xyl contents (% *w*/*w*) in the gizzard (Giz) and the ceca (Cec), (ii) acetate (Ace), butyrate (But), propionate (Pro) and total short-chain fatty acids (SCFA) contents in the ceca, (iii) nutrient digestibility parameters: apparent ileal digestibility (AID) and apparent total tract digestibility (ATTD) of organic matter (OM) starch (STA), and crude protein (CP), and (iv) animal growth parameters; body weight (BW), feed conversion ratio (FCR), average daily feed intake (ADFI) and average daily gain (ADG). Significance was set at *p* < 0.05.

The potential interrelationships between the investigated parameters were then examined (Figure 2 and Figure A3). Organic matter (OM) AID was negatively correlated with Ara-Cec and Xyl-Cec and with Ara-Ril. On the contrary, OM-ATTD presented positive correlations with Ara and Xyl contents in the gizzard (Giz) and the ceca, while it was negatively correlated with their ileal and total tract recoveries. Starch AID and ATTD were negatively correlated with Ara and Xyl contents in the gizzard and the ceca and were positively correlated with Ara and Xyl recovery in the excreta. The SCFA were negatively correlated with Ara and Xyl contents in the gizzard and the ceca but were positively correlated with Ara, Xyl recovery in the excreta (Rex), and starch ATTD. While unexpected, the positive correlation between Ara and Xyl total tract recovery and SCFA content was due to the maize-based DT, as those treatments presented high values for both sets of parameters. High SCFA loadings were positively correlated with improved animal growth. Improved animal growth was illustrated by high loadings of BW, ADFI, ADG, and low FCR loadings. Finally, the Ara and Xyl contents in the ceca were negatively correlated with animal growth.

## 4. Discussion

### 4.1. The Effect of NSPase on Carbohydrate Recovery and Oligosaccharide Profiles in the Upper GIT

#### 4.1.1. NSP Content in the Gizzard

The activity of dietary NSPases in the gizzard has been reported to be limited, mainly due to the acidic environment [42]. Nevertheless, cell wall degradation during the early stages of digestion [43] could set the scene for improved feed assimilation in the small intestine and the hindgut. Hence, the potential influence of dietary NSPases on carbohydrate content in the gizzard was investigated. The combination of xylanase and glucanase used in this study (NSPase) increased the arabinoxylan (AX) concentration in the gizzard but did not impact the A/X ratio (Table 3). This indicates the presence of higher levels of AX with similar structural characteristics in WE compared to WC. In addition, Gal and UA-containing NSP presented higher contents in the gizzard for WE. At the same time, WE presented a lower Glc content in the gizzard than WC. The differences in Glc content between WC and WE probably reflect differentiated starch retention in the gizzard, as affected by the enzyme, since non-starch Glc, such as cellulose, would be expected to behave similarly to other NSP. NSPase has been previously shown to influence the gizzard’s contents and empty weight, especially in whole wheat diets [17,42,44,45]. In addition, NSPase appeared to influence the type and level of feed components being retained in the gizzard. For example, the enzymatic degradation of the cell wall could have released entrapped nutrients, such as starch, thus facilitating their absorption in the small intestine. On the contrary, cell wall material has also been shown to accumulate in the gizzard, as previously reported [9,46]. The above observations were not present in the maize-based DT, as NSPase addition in ME did not impact the carbohydrate content. Still, further research is warranted to investigate the extent of the potential activity of NSPase in the gizzard.

#### 4.1.2. Xylanase Releases Soluble Oligosaccharides in the Small Intestine

Xylanase activity in the proximal GIT (gizzard, small intestine) is expected to have released arabinoxylan-oligosaccharides (AXOS). Non-digestible oligosaccharides, such as AXOS, are expected to accumulate in the ileum. Hence, the presence of soluble oligosaccharides in the ileum of the broilers was determined (Figure 1) to provide direct evidence of xylanolytic activity during feed digestion.

Hexose oligosaccharides (HexOS) detection in the ileum could be partly attributed to incomplete starch hydrolysis. For example, the presence of unabsorbed maltodextrins, mainly maltose and maltotriose, has previously been detected in the ileum of pigs [47]. The HexOS detected in WC, and WE presented longer chain lengths than the ones detected in MC and ME. Isolated wheat starch has been shown to be more rapidly digestible than maize starch [37]. Hence, none or only small differences between wheat and maize regarding the maltodextrins present at the end of the small intestine were expected. Therefore, maltodextrins alone are considered unlikely to account for the observed differences in HexOS profiles. It is suspected that HexOS represent (partly) a series of compounds with similar masses to maltodextrins. Wheat grains are known to contain fructans and fructo-oligosaccharides (FOS) [48], whose presence could further explain differences in HexOS profile between wheat-based and maize-based DT. Preliminary findings (data not shown) demonstrated the disappearance of most HexOS peaks after incubation with a combination of amyloglucosidase and endo- and exo-inulinase. The potential detection of FOS in the ileum presents great interest because these compounds are known for their prebiotic activity and can play an important role during hindgut fermentation [48,49].

Pentose oligosaccharides were detected in WE ileal digesta next to HexOS, and their release upon xylanase addition demonstrates the enzymatic degradation of (hetero)xylan to (A)XOS. Considering that both xylose and arabinose are pentoses, it was not possible to determine their relative ratio in each oligomer. Approximately 60–65% of the Xyl residues of wheat AX is unsubstituted, and these unsubstituted Xyl residues are distributed among Ara-substituted Xyl moieties as clusters of 2 to 5 consecutive residues [7,23,50,51]. Longer, unsubstituted xylan fragments would have either been further degraded by the xylanase or would have been adsorbed to cellulose and remained insoluble [52]. Consequently, the detected oligosaccharides consisting of 5 to 26 pentose units will contain both Xyl and Ara moieties in their structure, and they correspond to enzymatically released arabinoxylan-oligosaccharides (AXOS). Hence, it is demonstrated that dietary supplementation of xylanase led to the in situ release of AXOS in the ileum of broilers fed wheat-based diet. Pentose oligosaccharides below DP 6, ascribed as XOS, were recently detected in the jejunum of broilers fed wheat diets in the presence of xylanase as well as in the control treatment [53]. The presence of small, unsubstituted XOS (DP 2–6) could not be confirmed in the present study. AXOS DP > 5 were found to be the dominant oligomeric products of the enzymatic depolymerization of AX in the broiler’s small intestine by the supplemented xylanase. AXOS direct supplementation in broiler diets is reported to promote the growth of *Bifidobacteria* in the ceca [15,29]. Hence, the current findings strengthen the hypothesis that xylanase action in the upper GIT can generate oligosaccharides exhibiting prebiotic properties.

Enzyme addition in ME did not result in AXOS release. The recalcitrance of maize AX to xylanolytic activity can be attributed to its complex structure and low water-solubility [5,24,54,55]. For instance, substituents such as arabinose and glucuronic acid are known to hinder the activity of certain xylanases, especially the ones belonging to the GH11 family [56]. Indeed, the hindrance of GH11 xylanases toward maize AX has been reported in a previous in vitro study [55]. The potential oligosaccharide release by glucanase could not be confirmed in this study. This could be partly attributed to the low amount of β-glucan present in both wheat and maize [5]. Additionally, the high abundance of maltodextrins and FOS observed in the ileum could have potentially masked the presence of cello-oligosaccharides with the same mass. Hydrolysis of cell wall NSP, such as AX and cellulose, by xylanase and/or glucanase, is believed to have occurred in both wheat and maize but not necessarily resulting in oligosaccharide release. Therefore, partial cell wall degradation by NSPases may play an important role in reducing nutrient encapsulation by insoluble NSP [18,27].

#### 4.1.3. Implications of Oligosaccharide Release on NSP Ileal Recovery

NSP cannot be digested in the small intestine due to the lack of the necessary enzymes. Thus, they are expected to be fully recovered in the ileum. The high Ara, Xyl, Gal, and non-glucosyl NSP (NSG) ileal recoveries observed for all DT (Table 4) confirmed this assumption. In particular, more than 88% of the Xyl and approximately 100% of the Ara present in the diet were recovered in the ileum, regardless of DT. Ηigh ΑΧ accumulation in the broiler’s ileum [57,58] and complete ΑΧ recovery in the pig’s ileum [59] have been previously reported. However, insoluble digestibility markers, such as the one used in this study, may poorly estimate the transit of soluble feed components [60]. Furthermore, soluble and small feed particles exiting the ileum can enter the ceca, while insoluble, undigested feed components will be excreted [46,60]. Hence, the decreased Ara (6.9% lower) and Xyl (6.7% lower) ileal recoveries obtained in WE may imply a higher amount of soluble AX entering the ceca compared to WC. AXOS release by xylanase documented in WE (Figure 1) may have increased the proportion of soluble AX entering the ceca. The use of soluble digestibility marker is further needed to investigate this relationship [60].

Limited NSP fermentation and complete NSP recovery were expected for the small intestine due to short retention time, pH conditions, and small populations of *Lactobacilli* and *Clostridia* present [49]. Although WE presented higher acetate and succinate amounts than WC, the low absolute amounts of these metabolites further suggested limited fermentation in the ileum. *Lactobacilli* prefer the fermentation of maltodextrins formed during starch digestion compared to other oligosaccharides [61]. The marginal effect of NSPase on lactate formation observed in both wheat-based and maize-based DT may, consequently, not be directly related to NSP fermentation in the small intestine. This further strengthens the notion that the majority of NSP reaches the end of the small intestine undegraded by the microbiota, irrespective of enzyme treatment. It should be mentioned that direct AXOS provision to the diet increased lactate formation in the broiler’s ileum to a greater extent than xylanase treatment [62].

### 4.2. Enzyme Action Improves Nutrient Ileal Digestibility and Alters Their Use in the Hindgut

The possible impact of AX degradation in the upper GIT by NSPase on the ileal (AID) and the total tract digestibility (ATTD) of organic matter, starch, and protein was also investigated (Table 6).

Approximately 72.2% of the dietary organic matter (OM) was digested in the small intestine (AID), and an additional 1.8% of OM was fermented in the broiler’s hindgut in WC. Enzyme treatment (WE) increased OM-AID. Yet, the OM-ATTD did not increase further. This suggested that enzyme supplementation caused feed components that would have otherwise been fermented in the hindgut to be digested already in the ileum. Such observations were not applicable for the maize-based DT.

The bulk of starch (94.8–97.5%) was digested in the small intestine (AID) in all DT. Still, an additional 1.2% and 1.0% starch was fermented in the hindgut in WC and MC, respectively. It can be argued that part of the starch fraction escaping digestion disappeared through microbial fermentation in chicken’s hindgut, as previously mentioned for both pigs and poultry [63,64,65]. Resistant starch fermentation will occur in the ceca, where only soluble compounds and small particles can enter [46,60]. Starch fermentation in the hindgut provides less energy to the animal than starch digestion in the ileum [66]. Enzyme supplementation in WE and ME increased starch AID by 2.6% and 0.4% compared to WC and MC, respectively, whereas the total tract digestibility did not exceed that of the control DT (WC, MC). These observations indicate that NSPase enabled an increased starch absorption in the ileum. This might be nutritionally beneficial as pronounced ileal starch digestion has been associated with improved broiler performance [67,68].

Crude protein (CP) AID was positively influenced by NSPase in the wheat-based but not in the maize-based diet (Table 6). This remark demonstrates the importance of NSPase inclusion in wheat-based diets. Conversely, the higher protein digestibility of maize-based diets compared to wheat-based diets previously reported [16,68] may have limited the impact of enzyme supplementation. In addition, the higher soybean meal inclusion in the maize-based diet compared to the wheat-based diet (Table 1) meant that maize protein contributed less than wheat protein to the total protein of the diet and could potentially mask any effect of NSPase.

The difference in starch AID between WC and WE was six-fold higher than the difference in starch AID between MC and ME. The marked effect of NSPase on starch and protein digestibility highlighted the importance of enzyme inclusion in wheat-based diets. NSPase promotes nutrient digestion in wheat-based DT partly by reducing the digesta viscosity [12,13,68,69]. However, NSPase also subtly improved starch AID in maize-based DT, where digesta viscosity is not a limiting factor. This indicates that viscosity reduction is not the only mechanism involved [69]. Nutrient encapsulation in the cereal cell wall matrix is expected to limit their digestion [47,70]. The degradation of the cell wall matrix by xylanase and glucanase followed by the release of encapsulated nutrients could have further improved the nutritional values of both wheat and maize [16,27,59].

### 4.3. Carbohydrate Fermentation Patterns in the Hindgut of Broilers

In the wheat-based diets, AX hindgut fermentation was attested by the decreased Ara and Xyl recoveries for the total tract compared to the ileum. Still, more than 80% Ara and 70% Xyl were excreted unutilized. NSPase addition in WE tended to lower the recovery values for both Ara and Xyl compared to control treatment WC and, in addition, decreased Ara and Xyl levels and increased SCFA formation in the ceca. In particular, NSPase increased the formation of acetate and butyrate in the ceca, in line with previous research [17,20,71]. These SCFAs can be used by the host as an additional energy source and promote gut health [15]. From our results, it is demonstrated that the hydrolysis of wheat AX and the formation of AXOS by xylanase in the ileum in WE promoted AX fermentability by the microbiota in the ceca. Furthermore, the higher A/X ratio in the ceca in WE suggests that especially highly substituted AX fragments remain unfermented.

In contrast to the wheat-based DT, limited Xyl and Ara fermentation was observed in both MC and ME (Table 4). The recovery of both monosaccharides in excreta was approximately 100%, suggesting that maize AX is excreted virtually untouched. The insolubility of maize AX [5] was expected to result in a low proportion of AX entering the ceca. This was demonstrated in our study by the combination of low Xyl amount (<0.1% *w*/*w*) present in the ceca and high Xyl total tract recovery in MC and ME (Table 4). Similar to Ara and Xyl, also UA was excreted undegraded. This may reflect the structural complexity of maize glucuronoarabinoxylan, hindering xylanase activity and resulting in poor fermentability [50,54]. It should be noted that the higher soybean meal inclusion in maize-based DT compared to wheat-based DT could have impacted the estimation of mainly Ara and UA, as these monosaccharides are known to be abundant in soy NSP [54].

Gal was fermented to a greater extent (18%) in the wheat-based than in the maize-based DT. Additionally, UA fermentation was only observed in the wheat-based DT. These NSP components can derive from cereals [5], but most of Gal, Man, and UA present in the diet are expected to originate from pectins and hemicelluloses from soy [54]. It is not clear why the fermentability of these NSP sugars was more pronounced in the wheat-based DT. Possibly, microbiota stimulation due to AX fermentation in the wheat-based DT could have indirectly affected the use of other NSP.

Altogether, AX degradation by NSPase markedly improved AX fermentability and increased SCFA formation in WE. In contrast, NSPase treatment coincided with reduced SCFA contents in ME compared to MC. The subtle improvement in starch ileal digestibility observed in ME could have resulted in less (resistant) starch being available for fermentation. Hence, less available starch alongside poorly fermentable AX could explain the decrease in SCFA formation upon NSPase inclusion in the maize-based DT. Improved starch AID did not negatively influence SCFA formation in WE, probably due to a more pronounced NSP fermentation.

### 4.4. Interrelationships between Carbohydrate Fermentation, Nutrient Digestibility and Growth Parameters

Overall, the wheat-based DT was found more responsive to NSPase treatment than the maize-based DT regarding nutrient digestibility and NSP fermentability. The potential interrelationships between the investigated parameters could reveal how NSPases may affect the various biochemical and physiological responses in broilers. A schematic summary of how NSPases may have influenced the use of wheat-based and maize-based DT was based on PCA analysis (Figure 2).

Organic matter (OM) ATTD presented positive correlations with Ara and Xyl contents in the gizzard (Giz) and the ceca (Cec) and indicated the important role of AX in hindgut fermentation. The importance of AX fermentation in the ceca to produce SCFA was further attested by the negative correlations between SCFA and Ara and Xyl contents in the gizzard and the ceca. Moreover, total SCFA acetate, butyrate, and propionate correlated positively with BW, ADFI, and ADG and negatively with FCR. It seems that the activity of xylanase and the consequent AXOS formation in the proximal GIT (Figure 1) boosted the bacterial metabolism in the ceca, which in turn coincided with improved animal growth. This is in accordance with studies reporting the improved performance of wheat-fed broilers upon NSPase addition [12,16,17,20].

The above responses were seen for the wheat-based DT but not for the maize-based DT. The latter exhibited poor AX fermentability and low Ara, Xyl contents in the ceca. Despite that, both maize-based DT exhibited high SCFA contents and pronounced starch digestibility and growth parameters. Hence, the reverse correlations between starch AID and Ara and Xyl contents and the positive correlations of starch ATTD with SCFA and Ara and Xyl total tract recoveries could be explained. Limited maize AX fermentability meant that NSPase supplementation in maize-based diets could not have improved animal growth by a prebiotic mechanism. Moreover, the lower contribution of maize AX to digesta viscosity compared to wheat AX [5,69] could explain the more subtle impact of the enzymes on nutrient digestibility in maize-based DT. Conversely, the NSPase ability to depolymerize AX in wheat diets, thus promoting its fermentability while simultaneously facilitating nutrient digestion, emphasizes the importance of NSPase supplementation in wheat-based diets.

## 5. Conclusions

This study exhibited the enzymatic activity of dietary NSPase in the broiler’s upper gastrointestinal tract by the recognition of arabinoxylan degradation products as oligomers (AXOS) present in the ileum. The beneficial effect of dietary NSPase addition for broilers was dependent on the cereal type and level in the diet. This was affirmed by the more pronounced impact of the NSPase on the wheat-based diet and highlighted the different mechanisms at play for wheat and maize. NSPase promoted nutrient digestibility, especially that of starch and protein in the small intestine, and improved NSP fermentability in the hindgut in the wheat-based diet. The pronounced NSP fermentability might partly be attributed to the in situ formation of AXOS in the broiler’s upper GIT. The direct detection of oligosaccharides with prebiotic potential further established the link between dietary xylanase and pronounced microbial fermentation.

## Figures and Tables

**Table 1:** Ingredient composition (*w*/*w*% as-fed) of wheat-based and maize-based diets for the starter (day 0 to 10), grower (day 10 to 20), and finisher (day 20 to 28) phases.

Ingredient (%)	Wheat-Based			Maize-Based		
	Starter	Grower	Finisher	Starter	Grower	Finisher
Wheat	49.4	58.8	65.9	-	-	-
Maize	10.0	5.0	-	57.3	59.6	59.1
Soybean Meal 48CP ^1^	24.4	19.5	17.0	27.2	24.3	24.3
Toasted Soybeans	10.0	10.0	8.0	10.0	10.0	8.0
Soybean Oil	1.4	2.4	4.3	0.6	1.7	3.9
Monocalcium phosphate	1.4	1.3	1.0	1.5	1.4	1.2
Limestone	1.4	1.3	1.1	1.4	1.2	1.1
DL-Methionine	0.4	0.3	0.2	0.4	0.3	0.3
L-Lysine HCl	0.3	0.3	0.3	0.3	0.3	0.2
Salt	0.2	0.2	0.3	0.2	0.2	0.3
Na-Bicarbonate	0.3	0.3	0.2	0.3	0.3	0.2
L-Threonine	0.2	0.1	0.1	0.2	0.1	0.1
L-Valine	0.1	0.1	0.1	0.2	0.1	0.0
Coccidiostat	Sacox ^2^	Sacox	-	Sacox	Sacox	-
Premix Article ^3^	0.5	0.5	0.5	0.5	0.5	0.5
Diamol ^4^	-	-	1.0	-	-	1.0
Total	100.0	100.0	100.0	100.0	100.0	100.0

^1^ CP: Crude protein. ^2^ Provided by Huvepharma NV, Berchem, Belgium. ^3^ Providing per kg of diet: vitamin A (retinyl acetate), 10,000 IU; vitamin D_3_ (cholecalciferol), 2500 IU; vitamin E (dl-α-tocopherol acetate), 50 mg; vitamin K_3_ (menadione), 1.5 mg; vitamin B_1_ (thiamine), 2.0 mg; vitamin B_2_ (riboflavin), 7.5 mg; niacin, 35 mg; D-pantothenic acid, 12 mg; vitamin B_6_ (pyridoxine-HCl), 3.5 mg; vitamin B_12_ (cyanocobalamine), 20 µg; folic acid, 1.0 mg; biotin, 0.2 mg; choline chloride, 460 mg; Fe (FeSO_4_.H_2_O), 80 mg; Cu (CuSO_4_.5H_2_O), 12 mg; Zn (ZnO), 60 mg; Mn (MnO), 85; I (Ca(IO_3_)_2_), 0.8 mg; Co (Co_2_CO_3_(OH)_2_), 0.77 mg; Se (Na_2_O_3_Se), 0.15 mg. ^4^ Used as acid-insoluble ash (AIA) digestibility marker.

**Table 2:** Chemical composition (*w*/*w*% dry matter basis) and total sugar content of wheat-based and maize-based finisher diets.

Composition (%)	Wheat-Based	Maize-Based
Dry matter (% as-is)	90.3	89.5
Crude protein (N × 6.25)	20.5	20.7
Ash	5.9	6.5
Acid-insoluble ash (AIA)	0.96	0.94
Fat	ca 12.3 ^1^	ca 16.8 ^1^
Total carbohydrates	61.3	56.0
Starch	40.4	37.4
NSP ^2^	21.0	18.6
Glc ^3^	51.6	47.3
Non-glucosyl NSP (NGP) ^4^	9.8	8.7
Ara	2.1	1.8
Xyl	2.9	1.6
Gal	2.2	2.8
Uronic acid	1.9	1.7
A/X ^5^	0.7	1.1

^1^ Not determined, value calculated by difference (fat = dry matter − (crude protein + ash + total carbohydrates)). ^2^ Non-starch polysaccharides (NSP): residual amount between total carbohydrates and starch. ^3^ Glc: total glucose content.^4^ Non-glucosyl NSP (NGP): sum of all monosaccharides (incl. Man, Rha, Fuc), except Glc. ^5^ A/X: arabinose/xylose molar ratio.

**Table 3:** Effect of the enzyme (E) on the monosaccharide content (% *w*/*w* dry matter basis) in the gizzard and ceca of broilers fed wheat-based (WC, WE) (*n* = 6) and maize-based (MC, ME) (*n* = 6) DT.

**Gizzard**								
**% *w*/*w***	**WC**	**WE**	**SEM ^1^**	***p*-Value ^2^**	**MC**	**ME**	**SEM**	***p*-Value**
Ara	5.29	5.80	0.16	0.043	2.68	2.70	0.08	0.861
Xyl	7.80	8.90	0.35	0.051	3.00	2.65	0.13	0.093
Glc	38.49	34.46	0.96	0.014	33.73	34.95	0.55	0.145
Gal	3.52	3.84	0.05	0.001	2.67	2.54	0.06	0.174
UA	2.97	3.28	0.07	0.010	3.48	3.40	0.07	0.417
Total	59.62	58.05	0.82	0.203	46.68	47.27	0.46	0.393
NGP	21.13	23.59	0.58	0.013	12.95	12.31	0.33	0.198
A/X ^3^	0.68	0.65	0.01	0.230	0.90	1.03	0.04	0.062
**Ceca**								
**% *w*/*w***	**WC**	**WE**	**SEM**	***p*-Value**	**MC**	**ME**	**SEM**	***p*-Value**
Ara	0.31	0.24	0.02	0.059	0.22	0.19	0.01	0.066
Xyl	0.58	0.23	0.10	0.036	0.10	0.08	0.01	0.217
Glc	6.19	7.72	0.98	0.295	6.18	5.54	0.73	0.553
Gal	1.42	1.44	0.09	0.918	1.12	1.12	0.04	0.997
UA	1.22	1.26	0.04	0.563	1.00	0.88	0.06	0.217
Total	11.41	12.50	1.07	0.488	10.31	9.35	0.74	0.382
NGP	5.22	4.77	0.26	0.248	4.14	3.81	0.10	0.036
A/X	0.64	1.11	0.09	0.005	2.40	2.51	0.19	0.689

^1^ Standard error of the mean. ^2^ Estimated by ANOVA with enzyme addition (E) as a factor. ^3^ A/X: arabinose/xylose molar ratio.

**Table 4:** Effect of the enzyme (E) and sampling site (S) on Ara, Xyl, Gal, uronic acid (UA), and Non-glucosyl NSP (NGP) recovery (%) in the ileum and total tract of broilers fed wheat-based (WC, WE) (*n* = 5–6) and maize-based (MC, ME) (*n* = 5–6) DT.

Dietary Treatment (DT)	NSP Recovery %									
	Ara		Xyl		Gal		UA		NGP	
	Ileum	Total Tract	Ileum	Total Tract	Ileum	Total Tract	Ileum	Total Tract	Ileum	Total Tract
WC	107.97 ^a^	83.85 ^b^	94.82 ^a^	74.74 ^b^	104.47 ^a^	57.31 ^b^	73.55 ^a^	59.96 ^b,^*	94.81 ^a^	68.82 ^b^
WE	101.02 ^a^	80.22 ^b^	88.50 ^a^	70.29 ^b^	102.71 ^a,^*	58.77 ^b^	74.36 ^a^	62.49 ^b^	90.14 ^a^	68.00 ^b^
SEM ^1^	2.12		2.20		1.42 (* 1.57)		1.25 (* 1.39)		1.70	
	**Model established *p*-Values**									
E	0.022		0.025		0.996		0.193		0.125	
S	<0.001		<0.001		<0.001		<0.001		<0.001	
E × S	0.442		0.675		0.325		0.548		0.274	
MC	99.3	104.13	96.73	102.17	99.04 ^a^	67.63 ^b^	101.43	96.56	99.02	85.96
ME	105.85	109.47 *	102.16	107.88 *	107.54 ^a,^*	66.91 ^b,^*	96.02	101.66	99.83	92.98
SEM	3.92 (* 4.34)		3.10 (* 3.42)		2.82 (3.12)		3.43		3.53	
	**Model established *p*-Values**									
E	0.214		0.112		0.194		0.965		0.281	
S	0.743		0.111		<0.001		0.912		0.011	
E × S	0.665		0.977		0.143		0.143		0.390	

^1^ Standard error of the mean, for *n* = 6. * In case of missingness (*n* = 5), the adjusted SEM value is presented between brackets. ^a,b^ recovery values of the same NSP measured in the ileum and excreta within cereal type not sharing common notation differ significantly (*p <* 0.05).

**Table 5:** Effect of the enzyme (E) on acetate, lactate and succinate content in the ileum, and acetate, butyrate, propionate, isobutyrate, isovalerate, and total short-chain fatty acids (SCFA) content (μmol/g dry matter basis) in the ceca of broilers fed wheat-based (WC, WE) (*n* = 6) and maize-based (MC, ME) (*n* = 5–6) DT.

Dietary Treatment (DT)	Ileum (μmol/g)			Ceca (μmol/g)					
	Acetate	Lactate	Succinate	Acetate	Butyrate	Propionate	Isobutyrate	Isovalerate	Total SCFA ^2^
WC	2.52	129.42	3.30	172.66	53.12	11.02	1.38	1.51	239.69
WE	9.47	250.20	7.59	250.94	73.08	11.15	1.32	1.54	338.03
SEM ^1^	1.34	56.74	1.01	18.70	6.13	0.75	0.13	0.14	24.91
	**Model established *p*-Values**								
E	0.004	0.163	0.013	0.014	0.044	0.906	0.728	0.881	0.019
MC	9.95	239.87	9.47	354.47	78.46	31.43	3.38	4.26	472.42
ME	7.88	145.51	7.83	287.77	59.29	23.60	2.70	3.85	378.16
SEM	1.05	30.95	1.39	19.57	4.31	2.32	0.33	0.28	24.34
	**Model established *p*-Values**								
E	0.195	0.057	0.425	0.037	0.010	0.039	0.185	0.333	0.021

^1^ Standard error of the mean, for *n* = 6, except for isobutyrate and isovalerate in MC and ME, where *n* = 5. ^2^ Sum of individual SCFA in the ceca.

**Table 6:** Effect of the enzyme (E) and sampling site (S) on the apparent ileal digestibility (AID%) and apparent total tract digestibility (ATTD%) of organic matter, starch and crude protein of broilers fed wheat-based (WC, WE) (*n* = 6) and maize-based (MC, ME) (*n* = 5–6) DT.

Dietary Treatment (DT)	Organic Matter (OM)		Starch		Crude Protein (CP)	
	AID, %	ATTD, %	AID, %	ATTD, %	AID, %	ATTD, %
WC	72.17 ^b^	74.02 ^a^	94.76 ^b^	95.99 ^ab^	76.99	-
WE	75.31 ^a^	75.44 ^a^	97.35 ^a^	97.23 ^a^	81.17	-
SEM ^1^	0.38		0.45		0.64	
	**Model established *p*-Values**					
E	<0.001		<0.001		0.002	
S	0.019		0.232		-	
E × S	0.036		0.152		-	
MC	74.60	73.27	97.11 ^b^	98.15 ^a^	80.98	-
ME	74.75	73.48	97.48 ^a,b,^*	98.23 ^a^	78.21 *	-
SEM ^1^	0.57		0.23 * (0.26)		0.99 * (1.11)	
	**Model established *p*-Values**					
E	0.757		0.384		0.102	
S	0.033		0.001		-	
E × S	0.955		0.603		-	

^1^ Standard error of the mean, for *n* = 6. * In case of missingness, the adjusted SEM value (*n* = 5) is presented between brackets. ^a,b^ Values corresponding to the same measured parameter (OM, starch, CP) within cereal type not sharing common notation differ significantly (*p* < 0.05).

## Data Availability

The data presented in this study are available from the corresponding author on reasonable request.

## Abbreviations

DT	Dietary treatment	NGP	Non-glucosyl NSP
WC	Wheat control DT	AX	Arabinoxylan
WE	Wheat enzyme DT	AXOS	Arabinoxylan-oligosaccharides
MC	Maize control DT	HexOS	Hexose oligosaccharides
ME	Maize enzyme DT	XOS	Xylooligosaccharides
E	Enzyme	FOS	Fructo-oligosaccharides
S	Sampling site	OM	Organic matter
Ara	Arabinose	CP	Crude protein
Xyl	Xylose	AIA	Acid-insoluble ash
Glc	Glucose	AID	Apparent ileal digestibility
Gal	Galactose	ATTD	Apparent total tract digestibility
Man	Mannose	SCFA	Short-chain fatty acids
Rha	Rhamnose	BW	Body weight
Fuc	Fucose	FCR	Feed conversion ratio
UA	Uronic acids	ADFI	Average daily feed intake
A/X	Arabinose-to-xylose ratio	ADG	Average daily gain
NSP	Non-starch polysaccharides	MALDI-TOF-MS	Matrix-assisted laser desorption/ionization Time-of-flight mass spectrometry
NSPase	NSP-active enzyme		

## Appendix A

**Table A1:** Effect of the enzyme (E) on the body weight (BW at day (d) 28), feed conversion ratio (FCR), average daily feed intake (ADFI), and average daily gain (ADG) of broilers fed wheat-based (WC, WE) (*n* = 5–6) and maize-based (ME, MC) (*n* = 6) DT during the finisher period (days 24–28).

Dietary Treatment (DT)	BW (d28) (g)	FCR (g/g)	ADFI (g/d)	ADG (g/d)
WC	1290.00 *	1.62 *	123.31 *	76.38 *
WE	1370.00	1.50	130.17	87.10
SEM ^1^	18.80 (* 20.60)	0.03 (* 0.03)	4.00 (* 4.39)	3.30 (* 3.60)
	**Model established *p*-Values**			
E	0.021	0.018	0.281	0.059
MC	1311.67	1.53	124.55	81.82
ME	1353.92	1.50	129.45	86.73
SEM	18.22	0.03	3.04	3.29
	**Model established *p*-Values**			
E	0.136	0.498	0.285	0.320

^1^ Standard error of the mean, for *n* = 6. * In case of missingness, the adjusted SEM value (*n* = 5) is presented between brackets.

**Table A2:** Effect of the enzyme (E) on the monosaccharide content (% *w*/*w* dry matter basis) in the ileum and excreta of broilers fed the wheat-based (WC, WE) and maize-based DT (MC, ME) (*n* = 6).

**Ileum**								
**% (*w*/*w*)**	**WC**	**WE**	**SEM ^1^**	***p*-Value ^2^**	**MC**	**ME**	**SEM**	***p*-Value**
Ara	6.82	7.02	0.10	0.181	6.17	6.18	0.10	0.899
Xyl	9.24	9.49	0.19	0.367	5.68	5.76	0.08	0.495
Glc	18.25	15.27	0.66	0.009	14.38	14.49	0.44	0.857
Gal	7.59	7.91	0.23	0.349	10.12	9.80	0.51	0.660
UA	4.43	4.98	0.11	0.005	6.22	5.78	0.14	0.047
Total	48.75	47.21	0.54	0.073	45.51	44.88	0.48	0.383
NGP	30.50	31.94	0.52	0.080	31.13	30.39	0.61	0.411
A/X	0.74	0.74	0.01	0.914	1.09	1.07	0.02	0.559
**Excreta**								
**% (*w*/*w*)**	**WC**	**WE**	**SEM**	***p*** **-Value**	**MC**	**ME**	**SEM**	***p*** **-Value**
Ara	5.44	5.42	0.11	0.889	5.56	6.00	0.27	0.265
Xyl	7.64	7.34	0.27	0.439	5.47	5.73	0.20	0.375
Glc	13.87	12.62	0.59	0.162	10.35	10.35	0.34	0.991
Gal	4.36	4.57	0.08	0.090	6.26	6.22	0.20	0.889
UA	3.83	4.07	0.04	0.002	5.47	5.55	0.12	0.630
Total	37.12	36.06	0.44	0.122	34.96	35.95	1.03	0.513
NGP	22.90	23.45	0.33	0.276	24.61	25.60	0.77	0.386
A/X	0.73	0.74	0.01	0.582	1.02	1.04	0.02	0.286

^1^ Standard error of the mean. ^2^ Estimated by ANOVA with enzyme addition (E) as a factor.
